# Poly(Ethylene Glycol)-Poly(l-Alanine)/Hyaluronic Acid Complex as a 3D Platform for Understanding Cancer Cell Migration in the Tumor Microenvironment

**DOI:** 10.3390/polym13071042

**Published:** 2021-03-26

**Authors:** Jooyoung Sim, Hyun Jung Lee, Byeongmoon Jeong, Min Hee Park

**Affiliations:** 1 Center for Convergence Bioceramic Materials, Korea Institute of Ceramic Engineering and Technology, Cheongju 28160, Korea; jynjc1110@naver.com; 2 Department of Chemistry and Nanoscience, Ewha Womans University, Seoul 03760, Korea; serve04@ewhain.net

**Keywords:** tumor microenvironment, 3D matrix, multicellular spheroid, polymer complex, migration

## Abstract

Cancer progression and migration in the tumor microenvironment are related to cell types and three-dimensional (3D) matrices. Therefore, developing biomimetic tumor models, including co-culture systems and a tunable 3D matrix, could play an essential role in understanding the cancer environment. Here, multicellular spheroids using human adipose-derived mesenchymal stem cells (hADSCs) and breast cancer cells (MDA-MB-231) within the 3D matrix were used as a tumor microenvironment (TME) mimicking platform. The amphiphilic peptide block copolymer and hyaluronic acid (HA) formed a self-assembled structure, which provides a biocompatible 3D environment for the cells. Multicellular spheroids were formed on the optimized plate and were observed as cell migration from a spheroid within a 3D matrix, such as the invasive and metastatic cancer of TME. This study suggests a new 3D platform using polymer complexes and the importance of tumor complexities, including various cell types and microenvironments.

## 1. Introduction

Metastasis is responsible for about 90% of deaths from cancer [1]. Therefore, many researchers have developed in vitro tumor models of the most similar tumor microenvironment (TME) with patients to understand the tumor’s mechanism. TME is a complex environment consisting of tumor cells, tumor stromal cells, immune cells, and extracellular matrix (ECM) components such as collagen and hyaluronan [2]. The cells, soluble factors, molecules, ECM, and mechanical cues of TME can influence tumor growth, invasion, metastasis, host immunity and therapeutic effect [3]. Thus, different cellular and non-cellular components of TME are essential to mimic the in vitro tumor model.

Three-dimensional (3D) cell culture models, such as multicellular spheroids, closely mimic the main features of the structure, cellular assembly, hypoxia condition, and nutrient gradients in solid tumors [4]. Spheroid models are a particularly good platform for studying the invasion and metastasis of cancer cells by hypoxic signaling from primary tumors. For this reason, the spheroid models have demonstrated their potential for the development of anticancer drug screening platforms over time.

Collagen is a critical biomaterial in 3D models of cancer cell invasion and metastasis, that can be induced by adding different cell types, ratios, and other ECM components. As many tumors express collagen I in the ECM in vivo and increase, the collagen density correlates with tumor growth and metastasis [5,6,7]. The collagen fiber alignment and matrix stiffness affect the 3D cell migration of invasive cancer cells that play a role in tumor progression [8]. We previously reported biomimetic long range fibrous orientation by ionic interactions between (+)-charged amphiphilic peptide block copolymer and (-)-charged hyaluronic acid (HA) to mimic bundles of the nanofibrous structures in the ECM [9]. Beside, HA, as an ECM component that is similar to collagen, is a material that interacts with the transmembrane receptor CD44, which promotes cell adhesion and aggregation, and is used as a scaffold to induce cell assembly and to stimulate cancer cell migration and invasion [10,11]. Thus, this biomimetic 3D culture system with a nanofibrous structure can be used as a 3D scaffold to induce cancer cell migration to emulate TME, instead of the commercially available collagen and Matrigel systems.

Poly(ethylene glycol) (PEG)-polypeptides provide an amphiphilic character that can be induced and self-assembled into micelles, vesicles and nanofibers by controlling the molecular weight of the PEG and polypeptide. In addition, polypeptide accords diversity, including stereochemistry, hydrophilicity/hydrophobicity, a specific secondary structure such as a-helix, b-sheet and random coil [12,13,14,15,16]. Thermogels, such as PEG-polypeptide hydrogels, provide the biomimetic and cytocompatible 3D environment for the encapsulation of cells. They show the sol-to-gel transition by controlling temperature, then supply 3D space, high water content, and specific stiffness for the proliferation and differentiation of encapsulated cells [17,18,19].

In this study, we investigated the novel biomimetic 3D platform, consisting of a multicellular spheroid and PEG-poly(l-alanine) system which contained HA, mimicking the metastasis in TME of breast cancer for regulating cell migration. The polymer complex, through ionic interaction between amphiphilic polypeptide block copolymer and HA, was investigated to identify its possibility as a 3D matrix. Additionally, cell behavior from the multicellular spheroid embedded in the polymer complex was monitored as having a potential for mimicking the migration and invasion of cancer cells. A PEG-polyalanine-HA conjugated system (PHA) was used to provide a biocompatible 3D environment and cell adhesion site, an important parameter in TME, for inducing the cell migration [20].

## 2. Materials and Methods

### 2.1. Materials

Anhydrous *N,N*-dimethylformaide (Sigma-Aldrich, St. Louis, MO, USA), N-carboxy anhydrides of l-alanine (Onsolution, Seoul, Korea), α-amino-ω-methoxy-poly(ethylene glycol)s (PEG) (Mn = 2000 Da) (Pharmicell, Seoul, Korea), Diethyl ether (Daejung, Siheung, Korea), and Deuterium oxide (D_2_O), Trifluoroacetic acid-d (CF_3_COOD) (Sigma-Aldrich, USA) were purchased and used as received. Toluene (Daejung, Korea) was dehydrated by sodium. Chloroform (Daejung, Korea) was dried over magnesium sulfate. Sodium Hyaluronate (HA) (Mn = 14,800 Da) was purchased from Lifecore biomedical (Chaska, MN, USA). Human adipose-derived mesenchymal stem cells (hADSCs) and breast cancer cells (MDA-MB-231) were received from Cefobio (Seoul, Korea) and Korean Cell Line Bank (Seoul, Korea), respectively. RPMI-1640, fetal bovine serum (FBS) and penicillin/streptomycin (PS) were purchased from Hyclone (South Logan, UT, USA). 0.25% trypsin, 2.21mM EDTA, 1X sodium was purchased from Corning (Corning, NY, USA). 3-(4,5-Dimethylthiazol-2-yl)-2,5-Diphenyltetrazolium Bromide (MTT) and live/dead viability cytotoxicity kit were purchased from Invitrogen (Carlsbad, CA, USA).

### 2.2. Preparation of the mPEG-PA, HA and mPEG-PA/HA Complex Aqueous Solutions

The mPEG-PA (P) was synthesized by the ring-opening polymerization of the N-carboxy anhydrides of l-alanine, where α-amino-ω-methoxy-poly(ethylene glycol)s (PEG) was used as an initiator. The α-amino-ω-methoxy-PEG was dissolved in anhydrous toluene and the residual water was removed by azeotropic distillation. Anhydrous chloroform/*N,N*-dimethylformamide (5/1 *v*/*v*) and N-carboxy anhydrides of l-alanine were added to the reaction mixtures. The polymerization was carried out at 40 °C for 24 h under anhydrous nitrogen conditions. The polymer was purified by fractional precipitation by diethyl ether, and then the residual solvent was evaporated under vacuum. The polymer was dialyzed in water using a membrane (MWCO 2000) and freeze-dried. The final yield was about 60%. P and HA were used by dissolving in distilled water (DW) overnight at 12.0 wt.% and 1.0 wt.%, respectively. mPEG-PA/HA complex (PHA) was prepared by mixing P and HA in DW to a final concentration of 12.0 wt.% and 1.0 wt.%, respectively.

### 2.3. H-NMR and 2-Dimesional NMR Spectroscopy

The NMR spectroscopy (300 MHz FT-NMR Spectrometer; Varian, MA, U.S.A.) was studied to define the molecular weight of the polymer and the correlation between P (12.0 wt.%) and HA (1.0 wt.%). ^1^H-NMR spectrum of P was acquired at 25 °C in D_2_O and in CF_3_COOD, respectively. To gain further information about the interaction between P and HA, homonuclear 2-dimensional NMR spectrum was measured in D_2_O at 25 °C.

### 2.4. Fourier Transform Infrared Spectroscopy (FTIR)

Fourier transform infrared spectra (FTIR) were obtained in a wavenumber range from 4000 to 450 across six scans with a 0.15 resolution (FTIR spectrophotometer, Frontier, PerkinElmer). The P (12.0 wt.%) was prepared in gel state and freeze-dried to investigate at room temperature.

### 2.5. Dynamic Mechanical Analysis

The modulus of the P was investigated by strain-controlled rheometer, Anton Paar MCR 302 (Anton Paar GmbH, Graz, Austria) as a function of the temperature at 4–37 °C. The sample, which was in a fluidic state at 4 °C, was loaded between the parallel plates with a diameter of 25 mm and a gap of 0.5 mm. After loading the sample, the temperature was maintained at 4 °C for 10 min and raised to 37 °C at a heating rate of 2 °C/min and then maintained at 37 °C for 10 min. The experiment proceeded under a controlled frequency (1.0 rad/s) and 5% strain.

### 2.6. Dynamic Light Scattering(DLS) for Zeta-Potential

Zeta potentials of the P, HA, and PHA suspended in DW (1.0 wt%) were studied using a Zetasizer instrument (Zetasizer, ELSZ-2000, Otuska, Osaka, Japan).

### 2.7. Circular Dichroism (CD) Spectroscopy

Circular dichroism (CD) spectroscopy (CD-ORD Spectropolarimeter; Jasco Corporation, Japan) was measured as a function of concentration and temperature in the range of P, HA, and PHA aqueous solution (0.00625–0.1 wt.%) at 5–40 °C, respectively.

### 2.8. Scanning Electron Microscopy (SEM)

The P (12.0 wt.%), HA (1.0 wt.%), and PHA were quenched with liquid nitrogen at −196 °C and lyophilized, respectively. Scanning electron microscope (SEM) images were obtained by the SEM instrument (EM-30, COXEM, Daejeon, Korea). The sample was cross sectioned to obtain an SEM image.

### 2.9. Cell Culture

hADSCs and MDA-MB-231 were cultured in DMEM and RPMI-1640 cell culture medium containing 10% FBS and 1% PS, respectively. Cells were maintained in a humidified incubator at 37 °C with 5% CO_2_.

### 2.10. MTT Assay

The MTT assay was performed to investigate the cytotoxicity of P, HA, and PHA on breast cancer cells. MDA-MB-231 (1 × 10^4^) was seeded into a 96-well plate in 200 µL of cell culture medium and then incubated overnight. Cells were treated with varying concentrations (1.0–0.125 wt.%) of P, HA, and PHA (diluted in RPMI) for 24 h. Then, 50 µL MTT solution, prepared at 5 mg/mL in RPMI, was added to each well and incubated at 37 °C with 5% CO_2_ for 1 h. Sodium dodecyl sulfate (SDS) solution (0.5% SDS, 25 mM HCl, 90% isopropyl alcohol) was added to ensure total solubility of formazan crystals, and absorbance was recorded at 570 nm using the microplate reader (SpectraMax ABS, Molecular devices, San Jose, CA USA).

### 2.11. Live/Dead Assay

Cell viability of MDA-MB-231 in P, HA, and PHA was observed using a live/dead viability cytotoxicity kit. MDA-MB-231 (1 × 10^5^) were seeded in a glass-bottom culture dish with 2 mL of cell culture medium and then incubated overnight. Cells were treated with P, HA, and PHA (0.5 wt.% in cell culture medium) for 24 h. After 2 times of phosphate-buffered saline (PBS) washing, cells were incubated at room temperature for 30 min in a solution of 4 mM ethidium homodimer-1 (EthD-1) and 2 mM calcein AM with PBS. Labeled cells were then viewed under a Nikon Eclipse E600 fluorescence microscope and images were merged using EZ photo software. Live cells and damaged cells were stained with calcein AM (green) and EthD-1 (red), respectively.

### 2.12. D Cell Culture

The self-assembly of multicellular spheroids containing hADSCs and MDA-MB-231 were used to investigate cell behavior in the 3D matrix as a TME. hADSCs (A) and hADSCs/MDA-MB-231 (AM, 1:1 ratio) suspensions were seeded on an agarose-coated plate with 1 × 10^4^ cells/well. The agarose coated-plate was prepared by loading agarose solution (2% in PBS) in a 96-well plate and used after autoclave sterilization. The hADSC growth medium (Cefobio, Korea) was supplemented and cultured for 24 h in an incubator at 37 °C with 5% CO_2_. After embedding the A and AM in P and PHA aqueous solution on a 96-well plate, the plate was incubated at 37 °C with 5% CO_2_ to induce gelation of P (12.0 wt.%) and PHA. mPEG-PA/HA complex (PHA) was prepared by mixing P and HA in medium to a final concentration of 12.0 wt.% and 1.0 wt.%, respectively. Cell behavior of A and AM spheroids were observed at 0, 6, 12, and 24 h using an Axio Vert.A1 inverted microscope (Zeiss, Oberkochen, Germany).

## 3. Results and Discussion

### 3.1. Preparation of Thermosensitive Poly(l-Alanine) Hydrogel

**Figure 1:**
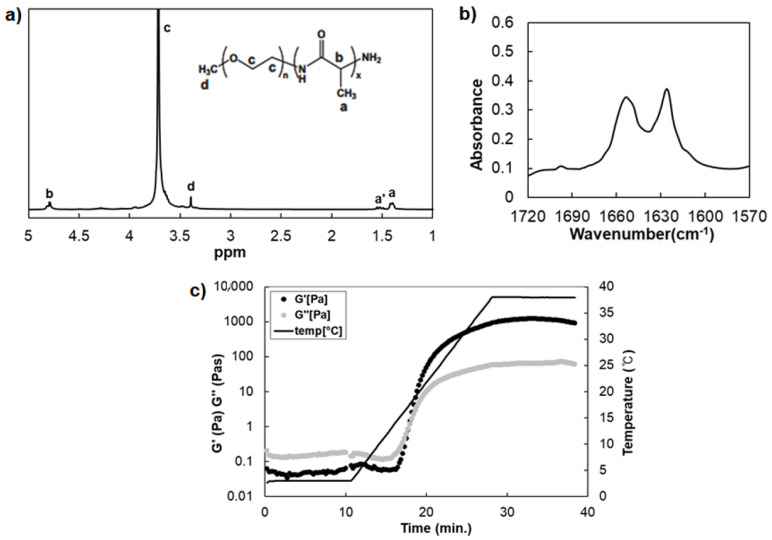
The characterization of mPEG-PA (P). (**a**) ^1^H-NMR spectra of P (25 °C in CF_3_COOD). (**b**) FTIR spectra of freeze-dried P. (**c**) Modulus of the P (12.0 wt.% in DW) as a function of temperature (4–37 °C).

The mPEG-PA (P) was synthesized by ring-opening polymerization of N-carboxy anhydrides of l-alanine in the presence of the α-amino-ω-methoxy-poly(ethylene glycol)s (PEG) [14,21]. The peaks of ^1^H-NMR spectra calculated the composition and molecular weight of P at 1.4–1.6 ppm (-CH_3_ of alanine), 3.3–3.5 ppm (-OCH_3_ of PEG end group), and 3.5–4.1 ppm (-OCH_2_CH_2_- of PEG) (Figure 1a). The molecular weight of each block of P was 2000–860 Daltons with the structure of (ethylene glycol)_44_-(l-alanine)_12_. The amide I band at 1570–1720 cm^−1^ in the FTIR spectra provides secondary structural information of a poly(l-alanine) [14]. A strong band at 1626 cm^−1^ and 1651 cm^−1^ of P indicated that the poly(l-alanine) is formed with β-sheet and α-helix secondary structures together (Figure 1b). The weak bands at 1695 cm^−1^ indicate an antiparallel-β-sheet of P [22]. The modulus of the P (12.0 wt.%) was measured while increasing the temperature by 2 °C/min from 4 °C to 37 °C and maintained at 37 °C. The modulus of the P shows the sol-to-gel transition as the temperature increased (Figure 1c). When the loss modulus (G”) is higher than the storage modulus (G’) at 4 °C, this means that the P is in a solution state and the opposite at 37 °C is in a gel state [23]. The G’ (about 1000–1300 Pa) increased rapidly more than 10,000-fold, and the intersection at 15 °C of G’ and G” indicates that a sol-to-gel transition of the aqueous polymer solution has occurred. These results mean that the nanostructure of poly(l-alanine) of P leads to a change in the macroscopic properties by a self-assembled structure of the secondary structure as a function of the temperature. In addition, the P provides a 3D matrix for cell cultures where a high level of modulus is required.

### 3.2. Characterization of PHA Complex

**Figure 2:**
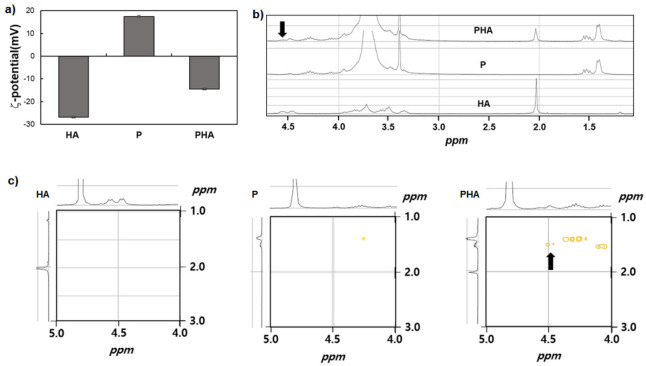
(**a**) Zeta-potential of HA, P, and PHA aqueous solutions. (1.0 wt.%) (**b**) ^1^H-NMR and (**c**) 2D-NMR spectra of HA, P, and PHA (25 °C in D_2_O).

mPEG-PA/HA complex (PHA) was prepared by mixing P and HA in DW to a final concentration of 12.0 wt.% and 1.0 wt.%, respectively. To identify the P and HA interactions, ζ-potential, ^1^H-NMR, 2D-NMR, and CD spectra of the PHA complex were investigated. The ζ-potential of P aqueous solution (1.0 wt.%) was +17.52 mV, which was reduced to −14.47 mV (PHA) by adding HA (Figure 2a). ^1^H-NMR spectra of the HA, P, and PHA were investigated at 25 °C (Figure 2b). After adding HA, the HA peak at 2.02 ppm became broader and shifted slightly, and a new broad peak at 4.56 ppm appeared in ^1^H-NMR spectra of PHA. The homonuclear 2D-NMR spectra of HA, P, and PHA at 25 °C were measured to assign and interpret the interactions between P and HA (Figure 2c). The black arrow in the 2D-NMR of PHA indicates the methyl/methine (-CH_3_/-CH-) coupling resulting from the complexation between the ammonium group of terminal alanine of P and the carboxylate groups of HA. These results support the chemical shift and appearance of a new peak in ^1^H-NMR spectra, and 2D-NMR indicates that the P interacted with HA by ionic complexation [9].

**Figure 3:**
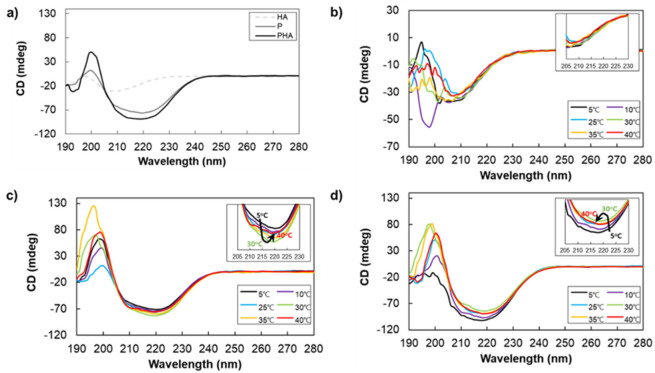
(**a**) The mean residue ellipticity of HA, P, and PHA aqueous solution (0.0125 wt.%) at 25 °C and CD spectra of (**b**) HA, (**c**) P, and (**d**) PHA according to temperature from 5 °C to 40 °C.

The poly(l-alanine), including α-helix, β-sheet, and random coil conformations, have their characteristic chiroptical properties in CD spectra [24,25]. The CD spectrum of the α-helix structures consists of a positive peak around 190-195 nm and two negative peaks at 205–210 nm and 215–225 nm. β-sheet structures show a positive peak around 192–200 nm and a negative peak at 210–220 nm. Random coil conformation shows a negative peak at 195–200 nm and a small positive peak at 215–220 nm [10]. The CD spectrum of polysaccharides is related to the molecular structure and conformation. In Figure 3a, the 210 nm band of HA could be attributed to the n-π* [26,27]. The CD peak of the P and PHA shows the β-sheet structure predominantly at 25 °C. However, the secondary structure of P changed from β-sheet to α-helix as the temperature increased (Figure 3c). In PHA, the β-sheet structure tends to dominate regardless of the temperature (Figure 3d). In addition, we found interesting results in which the changes in the CD patterns of P and PHA differ according to the temperature change. The CD values of P decreased while the temperature increased from 5 °C to 30 °C, and then increased with increasing temperature from 30 °C to 40 °C (Figure 3c). PHA, on the other hand, shows an opposite pattern; the CD value of PHA is increased when the temperature increased from 5 °C to 30 °C but there was a decreased peak when changing the temperature from 30 °C to 40 °C (Figure 3d). The CD values of HA showed no significant changes (Figure 3b). This trend is caused by a change in the secondary structure of P, a poly(l-alanine) polymer, through secondary structural change due to the attraction between P and HA. These results support that PHA has unique physical and chemical properties due to the interaction of P and HA [9].

### 3.3. Fabrication of Tumor Microenvironment Mimicking the 3D Matrix

**Figure 4:**
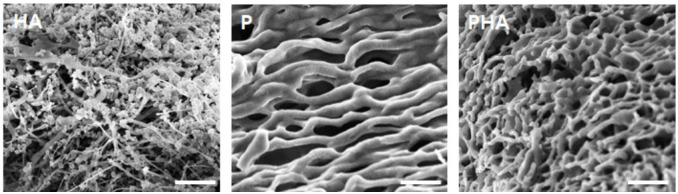
SEM images of freeze-dried HA (1.0 wt.%), P (12.0 wt.%), and PHA (12.0 wt.%) in DW. The scale bars are 50 μm.

The SEM examined the porous morphology of the freeze-dried HA (1.0 wt.% in DW), P (12.0 wt.% in DW), and PHA (12.0 wt.% in DW) to understand the 3D structure of hydrogel. Although the internal structure/morphology of the hydrogel after freeze-drying may differ from the natural state of the swollen hydrogel, it is still helpful to understand the details of the hydrogel 3D internal microstructure [28]. As shown in Figure 4, the PHA, compared with P, has a smaller pore size and a more compact sponge shape. This result confirmed that the structure of the hydrogel became denser with HA. This PHA structure can mimic the ECM of cancer cells, can make dense cellular aggregate in the normal cells, and promote cell migration and metastasis [29].

### 3.4. Cell Viability

**Figure 5:**
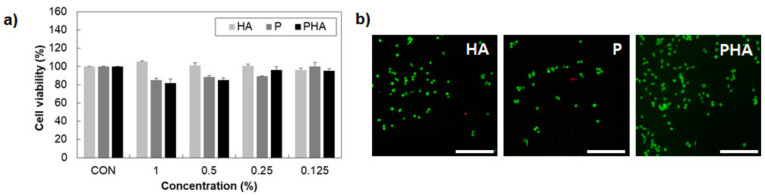
Cell viability of MDA-MB-231. (**a**) The MTT assay (n = 3) and (**b**) Live/Dead assay. The non-treated group was used as the control. Fluorescence microscopy images of MDA-MB-231 were obtained after 1 day of treatment with HA (0.042 wt.%), P (0.5 wt.%), and PHA (0.5 wt.%). The cells are stained using a Live (green)/Dead (red) kit, and the image showed green and red as live and dead cells, respectively. The scale bar is 200 μm.

MDA-MB-231, the breast cancer cell, was used to investigate cell cytotoxicity for HA, P and PHA. As shown in Figure 5a, HA, P, and PHA showed a cell viability of more than 80% at concentrations from 0.125 wt% to 1.0 wt% when compared to the non-treated group (CON). This indicates that HA, P and PHA can be used with the cell as the biocompatible material for mimicking the tissue microenvironment. This result was also confirmed by Live/Dead assay, using fluorescence microscopy to show that the cells are alive within HA, P, and PHA, where live and dead cells have appeared as green and red colors, respectively (Figure 5b).

### 3.5. Cell Behavior in 3D Matrix

**Figure 6:**
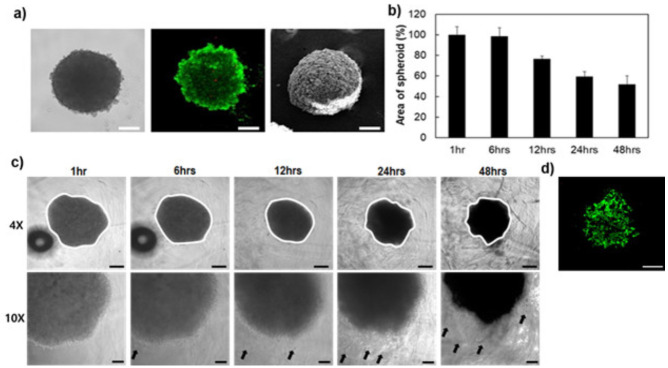
(**a**) The images of spherical assembly of AM using an inverted microscope, Live/Dead assay, and SEM. Cell behavior was measured (**b**) the spheroid area by Image J and (**c**) an inverted microscope at 1, 6, 12, 24, 48 h. (**d**) Live/Dead image of the spheroid at 48 h. The scale bar is 500 μm (4×) and 50 μm (10×), respectively.

The cancer research has been developed in vitro 3D models such as the multicellular tumor spheroid model (MCTS) [30,31,32,33] to mimic the tumor architectural features in biological processes. In this study, we engineered 3D tumor models using the cancer cell spheroid (AM), including MDA-MB-231 and hADSCs in polymeric scaffolds. The AM was prepared by inducing the self-assembly of hADSCs/MDA-MB-231 (AM, 1:1 ratio) on the agarose coated plate. The spherical assembly was observed using an inverted microscope and SEM after seeding (24 h) (Figure 6a). Most of the live cells indicate the green color, and the inside of spheroids showed minimal cell death by the lack of oxygen and nutrients, which indicates red color seeding (Figure 6a) [34,35,36]. In this study, the spheroids closely mimic the structural organization, hypoxia and nutrient gradients as the main features of solid tumors [4].

The behavior of cancer cells in the 3D matrix as mimicking the TME was examined by the area of the spheroid and cell movements from AM embedded in PHA using an inverted microscope. In PHA, the AM area was decreased by cell migration from the spheroid, indicating the black arrow (Figure 6b,c). MSCs enhance breast cancer cell growth, migration and invasion through the paracrine effects [37]. MSCs’ and breast cancer cells’ co-culture system also regulate EMT [38]. Thus, cells in AM had been induced motility by being co-cultured with hADSCs in the 3D matrix. Figure 6d shows the spheroid’s live and dead cells in PHA, indicating green and red color, respectively. This result means our system provides the biocompatible 3D environment to survive and migrate the cells during 48 h.

## 4. Conclusions

This study successfully prepared the self-assembled mPEG-PA/HA complex as a 3D matrix for mimicking the tumor microenvironment. The ion complexation between mPEG-PA and HA was analyzed for ζ-potential, ^1^H-NMR, 2D-NMR, and CD spectra. We also confirmed the biocompatibility of P and PHA. The 3D spheroids optimizing the agarose-coated plate observed spherical assembly and viability by optical microscopy, fluorescence microscopy and SEM. Finally, we induced cancer cell migration from a spheroid within a 3D matrix for mimicking the tumor microenvironment. In conclusion, the results of this study provide the new 3D platform with a tunable and compatible structure, and multicellular spheroids that can be used for understanding cancer invasion and metastasis in TME.

## Data Availability

Data available in a publicly accessible repository.
